# Total serum FGF-21 levels positively relate to visceral adiposity differently from its functional intact form

**DOI:** 10.3389/fendo.2023.1159127

**Published:** 2023-06-20

**Authors:** Lucilla Crudele, Oihane Garcia-Irigoyen, Marica Cariello, Marilidia Piglionica, Natasha Scialpi, Marilina Florio, Giuseppina Piazzolla, Patrizia Suppressa, Carlo Sabbà, Raffaella Maria Gadaleta, Antonio Moschetta

**Affiliations:** ^1^ Department of Interdisciplinary Medicine, University of Bari “Aldo Moro”, Bari, Italy; ^2^ Department of Biosciences and Nutrition, Karolinska Institutet, Huddinge, Sweden; ^3^ National Institute for Biostructures and Biosystems (INBB), Rome, Italy

**Keywords:** visceral obesity, HDL cholesterol, intact FGF21, waist circumference, vitamin D

## Abstract

**Objective:**

Increased Fibroblast Growth Factor-21 (FGF-21) circulating levels have been described in obesity. In this observational study, we analysed a group of subjects with metabolic disorders to unravel the putative link between visceral adiposity and FGF-21 serum levels.

**Methods:**

Total and intact serum FGF-21 concentration was measured with an ELISA assay respectively in 51 and 46 subjects, comparing FGF-21 levels in dysmetabolic conditions. We also tested Spearman’s correlations between FGF-21 serum levels and biochemical and clinical metabolic parameters.

**Results:**

FGF-21 was not significantly increased in high-risk conditions such as visceral obesity, Metabolic Syndrome, diabetes, smoking, and atherosclerosis. Waist Circumference (WC), but not BMI, positively correlated with total FGF-21 levels (r=0.31, p <0.05), while HDL-cholesterol (r=-0.29, p <0.05) and 25-OH Vitamin D (r=-0.32, p <0.05) showed a significant negative correlation with total FGF-21. ROC analysis of FGF-21 in prediction of increased WC, showed that patients with total FGF-21 level over cut-off value of 161.47 pg/mL presented with impaired FPG. Conversely, serum levels of the intact form of FGF-21 did not correlate with WC and other metabolic biomarkers.

**Conclusion:**

Our newly calculated cut-off for total FGF-21 according to visceral adiposity identified subjects with fasting hyperglycemia. However, waist circumference correlates with total FGF-21 serum levels but does not correlate with intact FGF-21, suggesting that functional FGF-21 does not necessarily relate with obesity and metabolic features.

## 1 Introduction

The Fibroblast Growth Factor-21 (FGF-21) is an atypical member of the Fibroblast Growth Factors (FGFs) family. FGF-21 lacks the conventional FGF heparin-binding domain (1, 2), therefore can diffuse away from its tissues of origin and functions as an endocrine hormone. In humans, FGF-21 production mainly occurs in the liver under the control of Peroxisome Proliferator-activated Receptor-alpha (PPAR-α), responsible for the downregulation of energy expenditure as a metabolic adaptation in response to prolonged starvation, high sugar intake, and dietary protein restriction (3). FGF-21 is a small protein comprising 181 amino acids that binds a receptor complex consisting of a classical FGF Receptor 1c (FGFR1c) and the co-receptor β-klotho (KLB), which is selectively expressed in metabolic tissues, such as white and brown adipose tissue (WAT and BAT), pancreas, muscle, heart, thymus where FGF-21 exploits several functions aimed to counteract metabolic derangement, metaflammation and stress damage (4). Fibroblast activation protein (FAP), a member of dipeptidyl peptidase-IV (DPP-IV) family, has been identified as the protease responsible for the proteolytic inactivation of human FGF-21 in blood, and cleavage of 10 amino acid residues at the C-terminus of the intact FGF-21 form, essential for its receptor binding and signaling activity (5). Indeed, Dunshee et al. showed that inhibiting FAP results in increased intact FGF-21 levels in cynomolgus monkeys (6). However, the exact mechanisms whereby FGF-21 mediates its pleiotropic actions have not been fully elucidated and information on FGF-21 gene expression in human tissues is limited since several discrepancies between animals and humans have been detected (7). A role for FGF-21 as a regulator of food intake and preferences in the central nervous system has been hypothesized (8); nevertheless, a wide interindividual variation of its serum level in the fasting state has been established (9). Physical exercise and lifestyle (9, 10), as well as drugs and dietary regimens (11), also influence FGF-21 levels as well as circadian variations have been detected with lower levels in the afternoon, likely due to increased free fatty acids (FFAs) plasma levels inducing the PPAR-α expression. Moreover, although direct correlations between FGF-21 levels and triglycerides (TG), glycemia, lipids, and insulin levels have been shown (12), the role of FGF-21 in obesity, diabetes, non-alcoholic fatty liver disease (NAFLD), Metabolic Syndrome (MetS), and coronary artery disease (CAD) is still debated (13–15). In fed state, FGF-21 cooperates with glucagon in promoting glucose synthesis, and stimulating glucose uptake in an insulin-independent manner (16). Paradoxically, it has been previously shown that circulating FGF-21 levels are already increased in these pathological conditions and several hypotheses have been formulated to explain this incongruity. It could either acting as a protecting factor against a metabolic overload or as a response to metabolic stress (7); for instance, a recent study found that FGF-21 increases in response to hypoxia and related oxidative stress in patients with obstructive sleep apnea, generally considered a component of MetS (17). Moreover, an FGF-21 resistance mechanism, similar to the insulin-resistance seen in diabetes, has been proposed (18).

From a clinical standpoint, assessing FGF-21 levels and understanding its function could make it a diagnostic and/or prognostic biomarker (19), as well as help discriminate patients responder vs non-responder to therapies, since Dutchark et al. showed that FGF-21 mediates thiazolidinediones antidiabetic actions (20). Furthermore, the pharmacological use of FGF-21 analogues has been proposed in the treatment of dyslipidemia, obesity, type 2 diabetes, and NASH (21–23).

In this study, we assessed intact and total FGF-21 levels in two cohorts of subjects with putative risk for MetS and obesity-related diseases with the goal of identifying the relationships between FGF-21 serum level and visceral obesity and its related complications.

## 2 Methods

### 2.1 Study population

Patients’ enrollment and clinical, biochemical, and instrumental evaluation were carried out at the Outpatient Clinic of Metabolic Disease of the Internal Medicine Division at Department of Interdisciplinary Medicine – “Aldo Moro” University of Bari (Italy). Patients with acute cardiovascular, renal, and hepatic failure, Inflammatory Bowel Disease and neoplastic diseases diagnosed less than 10 years before enrollment were excluded. The study was approved by the Ethics Committee of the Azienda Ospedaliero-Universitaria Policlinico di Bari (Bari, Italy) in accordance with the principles of the Declaration of Helsinki and all patients provided written informed consent prior to enrollment. In accordance with the approved Ethics Committee, only 18 years old or older patients were included in the study.

### 2.2 Clinical assessment

All participants underwent a detailed anamnesis and physical examination. Anthropometric assessment was performed using standardized procedures. The diagnosis of MetS was assessed in accordance to the National Cholesterol Education Program - Adult Treatment Panel III (NCEP-ATPIII) (24). Accordingly, visceral adiposity is defined as Waist Circumference (WC) > 102 cm for males and >88 cm for females. WC was measured at the midpoint between the inferior part of the 12th rib and the anterior-superior iliac crest. Body Mass Index (BMI) was computed as weight (Kg) divided by the height squared (sqm) and BMI values (Kg/sqm) between 25 and 29.9 and over 30 were classified as overweight and obesity, respectively. The population included patients affected by chronic high blood pressure (HBP) together with a chronic condition of hypertensive cardiopathy, diabetes, chronic renal failure, and chronic gastrointestinal diseases (gastroesophageal reflux, chronic gastritis, irritable bowel syndrome). Diabetes was defined according to HbA1c (percentage of glycosylated hemoglobin) ≥ 6.5%, fasting plasma glucose (FPG) ≥ 126 mg/dL (25) and/or treatment for diabetes (24). Hypertension was defined as systolic arterial blood pressure ≥ 130 mmHg, diastolic arterial blood pressure ≥ 85 mmHg and/or treatment with antihypertensive agents. B-mode ultrasonography with a 4- to 7-MHz linear array transducer was performed to detect carotid plaques in evaluation of atherosclerosis. Color and power Doppler were used to further delineate the plaque border (26).

### 2.3 Biochemical measurements

Morning blood samples were obtained after 12 hours of fasting from the antecubital veins. After blood clotting and centrifugation, serum was processed for analysis of biochemical markers of glucose and lipid metabolism. Liver, renal, thyroid, and inflammatory markers were also measured following standardized biochemical procedures. All biochemical measurements were performed in the ISO 9001 certified laboratories of the University Hospital of Bari.

Serum concentrations of total and intact forms of FGF-21 were assesed with ELISA assays and all measurements were carried out according to the manufacturers’ protocols. The Standard Curve was generated by plotting the absorbance versus human FGF-21 concentration for each standard provided by the supplier. Concentrations of unknown samples were determined by interpolation using this standard curve. Results are reported as concentration of FGF-21 (pg/mL) in the analysed samples. Total FGF-21 serum levels were measured with the RD191108200R Human FGF-21 ELISA (BioVendor Laboratory Medicine, Inc., Modrice, Czech Republic). Kit sensitivity was 7 pg/mL and upper limit of detection was 1920 pg/mL.

Intact FGF-21 was measured with the EDI Human Intact FGF-21 ELISA Kit (Epitope Diagnostics, Inc., San Diego, USA). The assay utilizes two selective antibodies that specifically bind to both the N- and C- terminus of human FGF-21. Kit sensitivity was 1.7 pg/mL and its upper limit of detection was 20000 pg/mL. All samples with values under or above the detectability limits were not considered for the analyses and those samples were excluded from both population studies.

### 2.4 Statistical analysis

All results are expressed as means ± standard error of the mean (SEM), in counts and percentages for categorical data. FGF-21 significant differences between two groups were determined by Mann-Whitney U test. The correlation between continuous variables was analysed using Spearman Correlation (r). All reported p-values were based on two-sided tests and compared to a significance level of 5%.

Cut-off point analysis was used to determine the optimal value of FGF-21 that differentiates patients who had increased WC. In particular, the crucial point was defined by the largest distance from the diagonal line of the receiver operating characteristic curve (ROC) (sensitivity x {1-specificity}). Empirical ROC curves were plotted along with calculation of the Area under the Curve (AUC) with 95% confidence intervals and one-sided upper p-values for null hypothesis AUC=0.5. Youden’s Index, or equivalently, the highest Sensitivity + Specificity, was used to determine the optimal cut-off. P-values of less than 0.05 were considered significant.

All analyses were performed using the NCSS 12 Statistical Software, version 12.0.2018 (NCSS, LLC Company, Kaysville, UT, USA) and GraphPad Prism, version 9.1.0 (GraphPad Software; San Diego, CA, USA).

## 3 Results

### 3.1 Clinical characterization of the study population

Fifty-one patients at their first assessment (24 males and 27 females), were recruited for evaluation of total FGF-21 serum levels from May 2015 to May 2016. Mean age of subjects was 61 ± 2 years. 30 patients (59%) were diagnosed with MetS, while 25 (49%) were diagnosed with Type 2 Diabetes. HBP was diagnosed in 36 patients (71%) and a carotid atherosclerotic plaque was detected in 14 subjects (27%). Total (cleaved and intact forms) FGF-21 mean serum level was 189.9 ± 38.1 pg/mL. Clinical characteristics of enrolled patients are shown in **Supplementary Table 1**.

No significant difference was found between males (242.9 ± 73.6 pg/mL) and females (142.6 ± 28.9 pg/mL). Data in **Table 1** display an increasing trend of FGF-21 levels (although not significant) when comparing patients with increased WC vs normal WC (NCEP-ATPIII criterion cut-off for WC), those affected by MetS vs patients without MetS, diabetic vs non diabetic, and patients with atherosclerosis vs patients without it.

**Table 1:** FGF-21 total serum level comparisons in individuals with and without high-risk conditions.

	NO	YES	p-value
Increased WC	144.1 ± 54.9	221.24 ± 52.8	ns
Metabolic Syndrome	137.4 ± 50.3	226.6 ± 54.1	ns
Diabetes	164.3 ± 45	216.5 ± 62.7	ns
Atherosclerosis	107.7 ± 39.5	211.9 ± 82.2	ns
Smoke	180.8 ± 40.3	230 ± 103	ns

Data is presented as mean ± SEM. Comparisons were performed by Mann-Whitney U test between patients with (YES) and without (NO) high-risk conditions. Metabolic Syndrome was diagnosed according to NCEP-ATPIII (National Cholesterol Education Program’s Adult Treatment Panel III). Positive NCEP-ATPIII criterion for Waist Circumference (WC) is >88 cm in females and >102 cm in males. For Diabetes, the criteria were: HbA1c (percentage of glycosylated hemoglobin) ≥ 6.5%, fasting plasma glucose (FPG) ≥ 126 mg/dL and/or treatment for diabetes. Hypertension was defined as systolic arterial blood pressure ≥ 130 mmHg, diastolic arterial blood pressure ≥ 85 mmHg and/or treatment with antihypertensive agents. B-mode ultrasonography with a 4- to 7-MHz linear array transducer was performed to detect carotid plaques in evaluation of atherosclerosis. Color and power Doppler were used to further delineate the plaque border. WC, waist circumference; ns, not significant.

### 3.2 Total FGF-21 serum level and visceral adiposity in high-risk conditions

**Figure 1:**
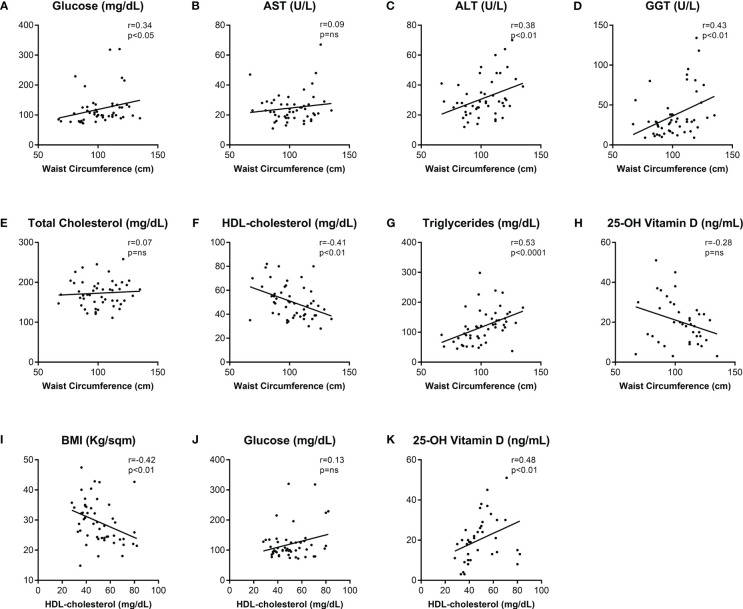
Correlations among clinical and biochemical metabolic biomarkers in the population study.Spearman’s correlations (r) of Waist Circumference **(A–H)** and HDL-cholesterol **(I–K)** with biochemical metabolic variables. p-values <0.05 were considered significant. Abbreviations: AST, aspartate transaminase; ALT, alanine transaminase; GGT, gamma-glutamyl transpeptidase; HDL, high-density lipoprotein; BMI, body mass index; ns, not significant.

Given the role of visceral obesity in determining dysmetabolism and the strong interplay among several biochemical variables and patients’ metabolic status, the relationships between WC and glucose and lipid metabolism biomarkers were assessed (**Figure 1**). Statistical analyses showed that there was a strong positive correlation between WC and FPG (r=0.34, p<0.05) and TG (r=0.53; p<0.001) levels. While total cholesterol did not correlate with WC, a remarkable inverse correlation between HDL cholesterol and WC was observed (r=-0,4, p<0.01). Since HDL-cholesterol levels reflect patients’ metabolic disorders and significantly correlates with obesity, correlation analyses between HDL and BMI, WC, and Vitamin D were also performed. HDL-cholesterol levels showed a negative significant correlation with both BMI and WC. Intriguingly, a positive significant correlation was found between HDL-cholesterol and 25-OH Vitamin D (r=0.48, p<0.01).

**Figure 2:**
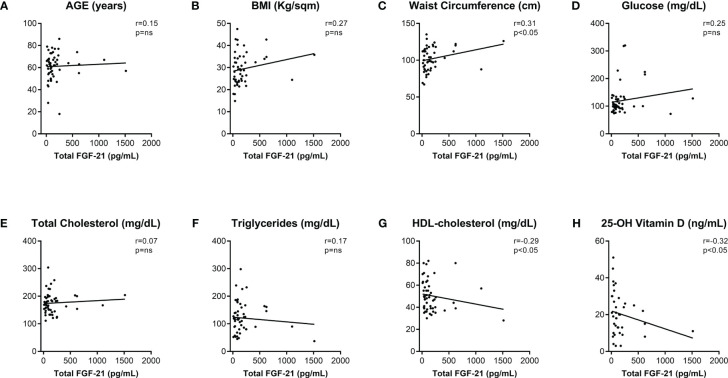
Correlations of total FGF-21 serum level with clinical and biochemical metabolic biomarkers. Total FGF-21 Spearman’s correlations (r) with clinical **(A–C)** and biochemical **(D–H)** metabolic variables metabolic variables. p-values <0.05 were considered significant. BMI, body mass index; HDL, high-density lipoprotein; ns, not significant.

In order to study the relation between patients dysmetabolism and FGF21 levels, serum total FGF-21 levels were correlated with other clinical and biochemical serum biomarkers. Age was not associated to total FGF-21 levels in the studied cohort (**Figure 2A**). Strikingly, while BMI did not correlate with total FGF-21 (**Figure 2B**), increase of WC directly reflected total FGF-21 levels (**Figure 2C**; r=0.31, *p*<0.05). Glucose, total cholesterol, and TG levels did not correlate with serum total FGF-21 (**Figures 2D–F**). Finally, an inverse relation between FGF-21 and both HDL-cholesterol (r=-0.29, *p*<0.05) and 25-OH Vitamin D (r=-0.32, *p*<0.05) were uncovered (**Figures 2G, H**).

### 3.3 Detection of FGF-21 cut-off value for prediction of visceral obesity

**Figure 3:**
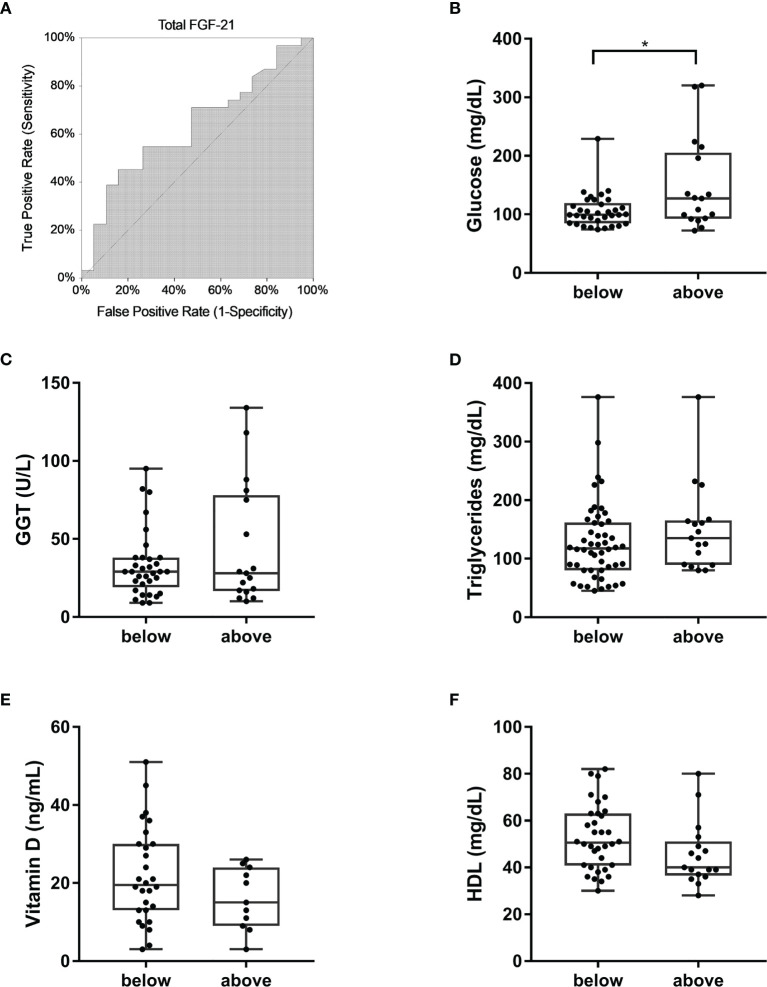
Total FGF-21 in prediction of visceral obesity and diabetes. **(A)** ROC curve of total FGF-21 for diagnosis of increased WC according to NCEP-ATPIII criterion. AUC=0.63, Z-value to test AUC>0.5=1.65, upper 1-sided *p*-value <0.05. Cut-off value=161.47 pg/mL. Comparisons of glucose **(B)**, GGT **(C)**, triglycerides **(D)**, Vitamin D **(E)**, and HDL-cholesterol **(F)** levels in patients with serum total FGF-21 level below and above the calculated cut-off value. *p-value <0.001. GGT, gamma-glutamyl transpeptidase; HDL, high-density lipoprotein.

Given the direct relationship between FGF-21 and WC, we performed a cut-off point analysis (AUC=0.63, p-value <0.05) to determine the serum total FGF-21 value able to discriminate patients who had visceral obesity according to NCEP-ATPIII WC criterion (**Figure 3A**). A cut-off value of 161.47 pg/mL was found, with a sensitivity of 45% and a specificity of 84%. Then, we categorized patients in two subgroups according to total FGF-21 serum level under (n=34) or above (n=17) the newly found cut-off value and a comparison of several biochemical parameters was performed. We found that FPG was significantly increased in those with increased FGF-21 levels (**Figure 3B**), discriminating people with fasting hyperglycemia (105.82 mg/dL vs 148.65 mg/dL). Also, there was an increase, although not significant, of GGT and TG levels (**Figures 3C, D**) in patients with total serum FGF-21 levels above the cut-off. Vitamin D and HDL were unchanged in the two groups (**Figures 3E, F**).

### 3.4 Intact FGF-21 serum level and visceral adiposity in high-risk conditions

**Figure 4:**
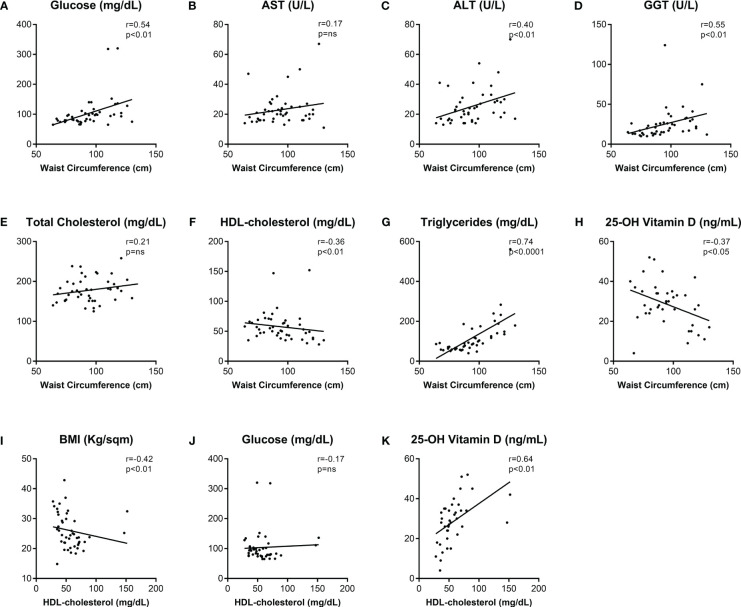
Correlations among clinical and biochemical metabolic biomarkers in another cohort of subjects. Spearman’s correlations (r) of Waist Circumference **(A–H)** and HDL-cholesterol **(I–K)** with biochemical metabolic variables. p-values <0.05 were considered significant. Abbreviations: AST, aspartate transaminase; ALT, alanine transaminase; GGT, gamma-glutamyl transpeptidase; HDL, high-density lipoprotein; BMI, body mass index; ns, not significant.

**Figure 5:**
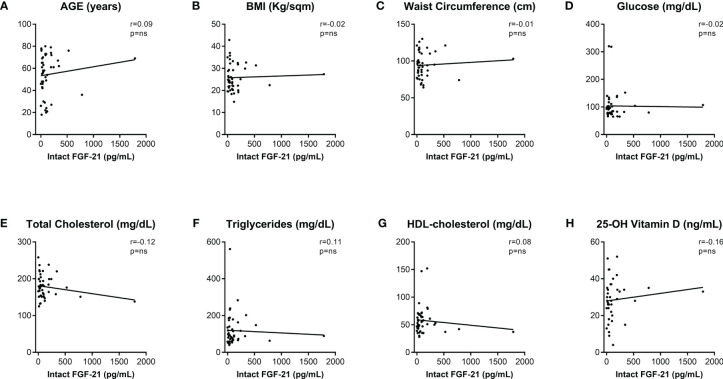
Correlations of intact FGF-21 serum level with clinical and biochemical metabolic biomarkers. Intact FGF-21 Spearman’s correlations (r) with clinical **(A–C)** and biochemical **(D–H)** metabolic variables. p-values <0.05 were considered significant. BMI, body mass index; HDL, high-density lipoprotein; ns, not significant.

Since only intact FGF-21 is able to cross-bind the co-receptor FGFR1c-KLB thus initiating the FGF-21 signaling cascade, and an assay determining total FGF-21 (both cleaved and intact forms) levels could overestimate the biological activity of this hormone (27), we measured intact FGF-21 levels in the serum of an additional population of forty-six patients at their first assessment (18 males, 28 females; mean age 54 ± 3 years) recruited from January to May 2023. Clinical characterization of these patients is included in **Supplementary Table 2**. The mean value of intact FGF-21 levels was 147.4 ± 42.2 (179.7 ± 99.8 in males, 126.7 ± 28.5 in females, p-value=not significant). Similar to the measurements of total serum FGF-21 levels, no significant differences in the FGF-21 intact form serum level were found in patients with high-risk conditions (**Table 2**). Moreover, also correlations between WC and glucose and lipid metabolism biomarkers (**Figures 4A–H**), as well as those between HDL and BMI, glucose, and Vitamin D were confirmed (**Figures 4I–K**) in this additional population. Similarly, age (**Figure 5A**), BMI (**Figure 5B**), glucose (**Figure 5D**), total cholesterol (**Figure 5E**) and triglycerides (**Figure 5F**) did not show any significant association with serum intact FGF-21 levels. Conversely, serum intact FGF-21 levels did not correlate any longer with WC (**Figure 5C**), HDL-c (**Figure 5G**) and Vitamin D (**Figure 5H**) levels. We also performed a ROC analysis for prediction of visceral adiposity by intact FGF-21 serum level finding a not-significant (p-value=0.18) AUC of 0.58.

**Table 2:** FGF-21 intact serum level comparisons in individuals with and without high-risk conditions.

	NO	YES	p-value
Increased WC	96.0 ± 30.5	209.1 ± 88.7	ns
Metabolic Syndrome	101.1 ± 28.1	219.4 ± 98.0	ns
Diabetes	98.9 ± 28.2	414.5 ± 97.7	ns
Atherosclerosis	70.6 ± 20.4	282.0 ± 143.2	ns
Smoke	178 ± 59.3	77.6 ± 24.4	ns

Data is presented as mean ± SEM. Comparisons were performed by Mann-Whitney U test between patients with (YES) and without (NO) high-risk conditions. Metabolic Syndrome was diagnosed according to NCEP-ATPIII (National Cholesterol Education Program’s Adult Treatment Panel III). Positive NCEP-ATPIII criterion for Waist Circumference (WC) is >88 cm in females and >102 cm in males. For Diabetes, the criteria were: HbA1c (percentage of glycosylated hemoglobin) ≥ 6.5%, fasting plasma glucose (FPG) ≥ 126 mg/dL and/or treatment for diabetes. Hypertension was defined as systolic arterial blood pressure ≥ 130 mmHg, diastolic arterial blood pressure ≥ 85 mmHg and/or treatment with antihypertensive agents. B-mode ultrasonography with a 4- to 7-MHz linear array transducer was performed to detect carotid plaques in evaluation of atherosclerosis. Color and power Doppler were used to further delineate the plaque border. WC, waist circumference; ns, not significant.

## 4 Discussion

FGF-21 is a peculiar stress-induced hormone-like molecule with pleiotropic functions regulating body energy homeostasis and lipid and glucose metabolism. In the last years, FGF-21 has drawn attention for its putative beneficial role in obesity-associated metabolic complications (28). Nevertheless, its complex mechanism of action is yet to be resolved, especially considering its tissue specificity and the current fragmentary translation of its physiological and pharmacological actions from rodents to humans. In this study, FGF-21 levels were analyzed in a cohort of patients with risk for dysmetabolic disorders.

Firstly, the hypothesis that FGF-21 serum level may predict or reflect a specific condition was tested. In line with previously published data, showing extremely variable FGF-21 levels, up to 250-fold variation even in healthy individuals (12, 29–31), our analysed cohort showed a heterogeneous amount of this hormone, regardless of sex and dysmetabolic conditions.

Interestingly, the level of total FGF-21 in our patients with diabetes and MetS were lower than the one previously shown in literature. While threshold values of 260 pg/mL and 270 pg/mL were proposed in differentiating patients with Insulin Resistance and MetS, respectively (32), in our sample mean values of circulating FGF-21 in diabetics and metabolic patients were lower and in line to another study that found median FGF-21 concentration of 239.9 pg/mL in diabetics subjects (33).

In the current study, an intriguingly significant correlation between circulating FGF-21 levels and WC, but not BMI and body weight, was established. This is in agreement with the concept that WC, rather than BMI, is a stronger indicator of Visceral Adipose Tissue (VAT) deposition and a stronger risk factor for the development of dysmetabolism-associated diseases. Here, with a ROC analysis we found that FGF-21 circulating level of 161.47 pg/mL serve as a discriminant value to identify people with visceral obesity. Moreover, individuals with FGF-21 level above this cut-off also presented with fasting hyperglycemia. Conversely, in a previous study by Mashili et al. (34) all measures of adiposity (BMI, waist circumference, and body fat percentage) were highly correlated with circulating total FGF-21 in patients with and without diabetes.

Intriguingly, FGF-21 mRNA expression in humans is almost exclusively limited to liver and its expression in adipose tissue (AT) is still debated (11, 35). Hepatic FGF-21 transcription is regulated by PPAR-α, PPAR-γ coactivator-1α (PGC-1α), retinoid acid receptor-related orphan receptor alpha (ROR-α), and high-carbohydrate diets, whereas in WAT its regulation depends on PPAR-γ, sirtuin 1 (SIRT1), and fast/refeeding regimens (16). Intriguingly, a difference in *mRNA* expression level between patients with and without obesity was found in VAT, but not in Subcutaneous AT (SAT) (11). Consequently, only VAT seems to be implied in FGF-21-regulated pathways in obesity. In this study, the direct correlations between WC and FPG, ALT, GGT, and TG, suggest that the VAT-derived adipokines and signaling factors could indeed drive diabetes onset and development and NAFLD pathogenesis, as previously reported (36, 37). In the analysed samples the inverse correlation between GGT and WC becomes stronger when FGF-21 levels increased, suggesting that FGF-21 is higher in patients with a more severe condition of fatty liver and impaired metabolism.

The inverse correlations between HDL-c levels and either WC or BMI confirmed what we have previously shown in a cohort of metabolic patients (38). HDL particles are implied in reverse cholesterol transport (RCT), conveying cholesterol from peripheral tissues to the liver, a crucial process to prevent atherosclerosis (39). In the current study, there was no significant increase in circulating FGF-21 level in patients with atheromatous plaque however FGF-21 inversely correlated with HDL. In line with previously reported data (12, 40, 41), this study shows not only an inverse relationship between FGF-21 and HDL-c but also a direct correlation between HDL-c and Vitamin D levels, as we previously reported (42), thus supporting the hypothesis that FGF-21-driven putative protective role against diabetes and obesity may depend on normal Vitamin D and HDL-cholesterol levels. For these reasons, for the first time, we went further and evaluated the relation between FGF-21 and 25-OH Vitamin D. In the analysed cohort, 25-OH Vitamin D showed a significant inverse correlation with FGF-21 levels. Vitamin D is mainly stored in AT, slowly released in the systemic circulation during fasting periods and regulates adipogenesis (43). Furthermore, this hormone is implicated in the modulation of genes involved in cholesterol homeostasis via the activation of Vitamin D Receptor (VDR) and its serum levels are negatively associated to higher WC and inflammation (44). A recent systematic review and meta-analysis has shown that Vitamin D may be considered an adiponectin secretagogue in patients with diabetes (45). Serum adiponectin levels are positively associated with 25-OH Vitamin D levels and HDL, and negatively correlates with inflammatory markers, FPG, TG, LDL (46). Vitamin D supplementation increased adiponectin serum levels in diabetic patients ameliorating HbA1c and BMI (47). Adiponectin is critical for FGF-21 glycemic and insulin sensitizing effects. Indeed, FGF-21 stimulates adiponectin secretion in rodents while adiponectin-knockout mice are refractory to changes in energy expenditure evoked by FGF-21 administration (48, 49). Significant inverse correlations between FGF-21 concentrations and adiponectin and HDL cholesterol were already observed (33) but if Vitamin D may explain, mediate or being a consequence of this relationship has never been explored. Vitamin D involvement in FGF-21 pathway should not be unexpected considering that 1,25-OH Vitamin D activates nuclear receptor VDR and increases FGF-23 production in bones (21). FGF-23 belongs to the same family of FGF-21 and acts as an endocrine nexus between hormones and mineral ions with the result of preventing hyperphosphatemia and hyper-vitaminosis. Consequently, pinpointing FGF-21 interaction with other hormones, such as adiponectin, may help our understanding about its involvement in metabolic pathways.

The FGF21 paradox lays its foundation on the finding that although counteracting metabolic derangement, high levels of this hormone are observed in obese and dysmetabolic subjects. It has been speculated that FGF-21 production could represent a response of the organism to cope with dysmetabolism caused by obesity and MetS (50). Consequently, the higher, although not significantly, total serum FGF-21 concentration in diabetics could potentially be the result of a compensatory mechanism to metabolic disturbances or tissue resistance to FGF-21 activity. In fact, previous findings about increased total serum FGF21 levels could putatively be ascribed to a coping mechanism trying to overcome its impaired biological function in obese patients according to two possible mechanisms: FGF21 receptors dysfunctional activation (51) and/or increased FAP enzymatic cleavage activity. Our results showing that intact FGF-21 levels are not correlated with waist circumference, Vitamin D and HDL-c, differently from total serum FGF-21 levels, suggests the fact that functional FGF-21 does not necessarily relate with obesity and metabolic features. This is in line with hypothesis that those subjects might have increased total FGF-21 but not functional FGF-21 that therefore does not predict the dysmetabolic conditions. Overall, from a clinical point of view, measurement of intact functional serum form of FGF-21 will help clinicians to identify those patients who could eventually benefit from therapies aiming to increase the hormone level in serum as well as treatments increasing endogenous functional level of FGF-2, using DPP-IV or FAP inhibitors. For instance, the association of fibrate and a FAP inhibitor may offer an oral therapy to augment endogenous FGF-21 actions (52) as well as sitagliptin, a DPP-IV inhibitor, has been found to inhibit FAP-activity and increase intact FGF-21 (53). Moreover, despite enduring controversy, lifestyle and pharmacological anamnesis should potentially be considered when characterizing patients since physical exercise and drugs have been suggested to affect FGF-21 levels.

In conclusions, our newly calculated cut-off of total FGF-21 according to visceral adiposity identified subjects with fasting hyperglycemia, and visceral adiposity appears to be responsible for increased levels of total FGF-21, although this does not necessarily translate into an increased hormonal biological activity, since levels of intact functional FGF-21 hormone are not related to abdominal obesity.

## Data availability statement

The raw data supporting the conclusions of this article will be made available by the authors, without undue reservation.

## Ethics statement

The studies involving human participants were reviewed and approved by Ethics Committee of the Azienda Ospedaliero-Universitaria Policlinico di Bari (Bari, Italy). The patients/participants provided their written informed consent to participate in this study.

## Author contributions

Conceptualization, AM; methodology-visualization, LC; software-formal analysis, LC; lab methodology, OG-I, MP, NS, MF, RG; investigation, LC, MF, GP, PS; resources, LC, RG, MF, AM; data curation, LC; writing—original draft preparation, LC, RG, MC; writing—review and editing, AM; supervision, AM; project administration, AM; funding acquisition, AM. All authors contributed to the article and approved the submitted version.
