# Supplementary material for: Total serum FGF-21 levels positively relate to visceral adiposity differently from its functional intact form
Source: Front Endocrinol (Lausanne). 2023 Jun 20;14:1159127. doi: 10.3389/fendo.2023.1159127 (PMC10319105; doi:10.3389/fendo.2023.1159127)
Supplement: Supplementary file 1 [file DataSheet_1.docx]

Supplementary Material

Total serum FGF-21 levels positively relate to visceral adiposity differently from its functional intact form.

**Lucilla Crudele^1†^, Oihane Garcia-Irigoyen^1,2†^, Marica Cariello^1^, Marilidia Piglionica^1^, Natasha Scialpi^1^, Marilina Florio^1^, Giuseppina Piazzolla^1^, Patrizia Suppressa^1^, Carlo Sabbà^1^, Raffaella Maria Gadaleta^1*^, Antonio Moschetta^1,3*^**

*** Correspondence:**[raffaella.gadaleta@uniba.it](mailto:raffaella.gadaleta@uniba.it), [antonio.moschetta@uniba.it](mailto:antonio.moschetta@uniba.it).

| **STUDY POPULATION** | **51 (24M:27F)** | |
| --- | --- | --- |
| Age (years) | 61±2 | |
| BMI (Kg/sqm) | 29±1 | |
| Waist circumference (cm) | 102±2 | |
| Glucose (mg/dL) | 120±8 (n.v.<100) | |
| HbA1c (mmol/mol) | 47±2 (n.v. 20-42) | |
| AST (U/L) | 24±1 (n.v.<34) | |
| ALT (U/L) | 31±2 (n.v. 10-49) | |
| GGT (U/L) | 37±4 (n.v.<73) | |
| Total Cholesterol (mg/dL) | 176±5 (n.v.<200) | |
| HDL-cholesterol (mg/dL) | 50±2 (n.v.>40 in males and >50 in females) | |
| LDL-cholesterol (mg/dL) | 98±5 (n.v.<160) | |
| Triglycerides (mg/dL) | 131±11 (n.v.<150) | |
| ESR (mm/hour) | 23±3 (n.v. 1-15) | |
| CRP (mg/L) | 4.9±0.7 (n.v.<2.9) | |
| 25-OH Vitamin D (ng/mL) | 20±2 (n.v.>30) | |
| Total FGF-21 (pg/mL) | 189.9±38.1 | |
| Metabolic Syndrome | 30 (16M:17F) – 59% | |
| WC positive criterion | 31 (19M:23F) – 61% | |
| BMI 25-30 | 9 (5M:4F) – 18% | |
| BMI >30 | 22 (12M:10F) - 43% | |
| Smokers | 14 (6M:8F) – 27% | |
| Arterial Hypertension | 36 (18M:18F) – 71% | |
| Atherosclerosis | 14 (5M:9F) – 27% | |
| Diabetes | 25 (12M:13F) – 49% | |
| Treatment for diabetes (n=24) | Metformin | 14 |
|  | Metformin + liraglutide | 2 |
|  | Metformin + vildagliptin | 1 |
|  | Metformin + sitagliptin | 2 |
|  | Metformin + Insulin | 2 |
|  | Insulin | 3 |
| Lipid lowering treatment (n=25) | Statins | 20 |
|  | Fibrates | 2 |
|  | Statins + fibrates | 3 |

**Supplementary Table 1. Clinical Characterization of the study population in which the total FGF-21 serum level was measured**. Metabolic Syndrome was diagnosed according to NCEP-ATPIII (National Cholesterol Education Program’s Adult Treatment Panel III). Positive NCEP-ATPIII criterion for Waist Circumference (WC) is >88 cm in females and >102 cm in males. For Diabetes, the criteria were: HbA1c (percentage of glycosylated hemoglobin) ≥ 6.5%, fasting plasma glucose (FPG) ≥ 126 mg/dL and/or treatment for diabetes. Hypertension was defined as systolic arterial blood pressure ≥ 130 mmHg, diastolic arterial blood pressure ≥ 85 mmHg and/or treatment with antihypertensive agents. B-mode ultrasonography with a 4- to 7-MHz linear array transducer was performed to detect carotid plaques in evaluation of atherosclerosis. Color and power Doppler were used to further delineate the plaque border. Data is presented as mean±SEM for numerical data, in counts and percentages for categorical data. Abbreviations: M, males; F, females; BMI, body mass index; HbA1c, glycosylated hemoglobin; AST, aspartate transaminase; ALT, alanine transaminase; GGT, gamma-glutamyl transpeptidase; HDL, high-density lipoprotein; LDL, low-density lipoprotein; ESR, erythrocytes sedimentary rate; CRP, C-reactive protein; n.v, normal values

| **STUDY POPULATION** | **46 (18M:28F)** | |
| --- | --- | --- |
| Age (years) | 54±3 | |
| BMI (Kg/sqm) | 26±1 | |
| Waist circumference (cm) | 94±3 | |
| Glucose (mg/dL) | 104±8 (n.v.<100) | |
| HbA1c (mmol/mol) | 42±2.6 (n.v. 20-42) | |
| AST (U/L) | 23±2 (n.v.<34) | |
| ALT (U/L) | 25±2 (n.v. 10-49) | |
| GGT (U/L) | 25±3 (n.v.<73) | |
| Total Cholesterol (mg/dL) | 178±5 (n.v.<200) | |
| HDL-cholesterol (mg/dL) | 58±4 (n.v.>40 in males and >50 in females) | |
| LDL-cholesterol (mg/dL) | 106±4 (n.v.<160) | |
| Triglycerides (mg/dL) | 118±13 (n.v.<150) | |
| ESR (mm/hour) | 21±3 (n.v. 1-15) | |
| CRP (mg/L) | 9.5±4 (n.v.<2.9) | |
| 25-OH Vitamin D (ng/mL) | 29±2 (n.v.>30) | |
| Intact FGF-21 (pg/mL) | 147.4±42.2 | |
| Metabolic Syndrome | 18 (8M:10F) – 39% | |
| WC positive criterion | 20 (7M:13F) – 43% | |
| BMI 25-30 | 10 (4M:6F) – 22% | |
| BMI >30 | 11 (6M:5F) - 24% | |
| Smokers | 14 (5M:9F) – 30% | |
| Arterial Hypertension | 23 (12M:11F) – 50% | |
| Atherosclerosis | 12 (3M:9F) –26 % | |
| Diabetes | 18 (8M:10F) – 40 % | |
| Treatment for diabetes (n=17) | Metformin | 11 |
|  | Metformin + semaglutide | 2 |
|  | Metformin + linagliptin | 1 |
|  | Insulin | 3 |
| Lipid lowering treatment | Statins | 13 |

**Supplementary Table 2. Clinical Characterization of the study population in which the intact form of FGF-21 serum level was measured**.
Metabolic Syndrome was diagnosed according to NCEP-ATPIII (National Cholesterol Education Program’s Adult Treatment Panel III). Positive NCEP-ATPIII criterion for Waist Circumference (WC) is >88 cm in females and >102 cm in males. For Diabetes, the criteria were: HbA1c (percentage of glycosylated hemoglobin) ≥ 6.5%, fasting plasma glucose (FPG) ≥ 126 mg/dL and/or treatment for diabetes. Hypertension was defined as systolic arterial blood pressure ≥ 130 mmHg, diastolic arterial blood pressure ≥ 85 mmHg and/or treatment with antihypertensive agents. B-mode ultrasonography with a 4- to 7-MHz linear array transducer was performed to detect carotid plaques in evaluation of atherosclerosis. Color and power Doppler were used to further delineate the plaque border. Data is presented as mean±SEM for numerical data, in counts and percentages for categorical data. Abbreviations: M, males; F, females; BMI, body mass index; HbA1c, glycosylated hemoglobin; AST, aspartate transaminase; ALT, alanine transaminase; GGT, gamma-glutamyl transpeptidase; HDL, high-density lipoprotein; LDL, low-density lipoprotein; ESR, erythrocytes sedimentary rate; CRP, C-reactive protein; n.v, normal values.
